# Hepatic transcriptomics reveals immuno-metabolic interactions in juvenile channel catfish (*Ictalurus punctatus*) after *Aeromonas hydrophila* infection

**DOI:** 10.1186/s12864-026-12988-1

**Published:** 2026-05-28

**Authors:** Yesutor K. Soku, Miles D. Lange, Jason W. Abernathy, Nithin M. Sankappa, Craig A. Shoemaker, Karl Hayden, Linnea K. Andersen, Ida Phillips, Toufic Nashar, Temesgen Samuel, Abdelrahman Mohamed

**Affiliations:** 1 Department of Pathobiology, College of Veterinary Medicine, Tuskegee University, Tuskegee, AL USA; 2 Department of Biology, College of Arts and Sciences, Tuskegee University, Tuskegee, AL USA; 3 United States Department of Agriculture, Agricultural Research Service, Aquatic Animal Health Research Unit, Auburn, AL USA; 4 Oak Ridge Institute for Science and Education (ORISE), ARS Research Participation Program, Oak Ridge, TN USA; 5 North Carolina Veterinary Diagnostic System, Raleigh, NC USA

**Keywords:** *Aeromonas hydrophila*, Channel catfish (*Ictalurus punctatus*), Hepatic response, Host-pathogen interaction, Immunometabolism, Liver, Transcriptomics, Aquaculture

## Abstract

**Background:**

In aquaculture, disease outbreaks occur amidst complex husbandry factors like feeding schedule and incidental injury. The liver, a central immunometabolic organ, integrates these cues, but the systems-level transcriptional mechanisms governing its response to concurrent stress are poorly defined in channel catfish. We investigated how feeding status and physical injury modulate the hepatic transcriptome during early *Aeromonas hydrophila* infection in channel catfish.

**Results:**

Juvenile channel catfish were assigned to four treatment groups reflecting combinations of feeding status (fed or fasted) with or without adipose fin‑clipping followed by immersion challenge with virulent *A. hydrophila*. Although the design captured interactions among these factors within the infected groups, it does not represent a full factorial structure as all infected groups were only compared to (fed with or without fin-clipping controls) and did not include the reciprocal (fasted with or without fin-clipping controls). Histopathology at 2, 4, and 8 h post-challenge (hpc) revealed progressive vascular congestion and immune infiltration primarily in fasted, unclipped fish. RNA-Seq analysis of liver tissue, focusing on genes that were differentially expressed across all timepoints when compared to fed with or without fin-clipping controls, showed marked transcriptional reprogramming. Under the present experimental and control framework, fasted fish showed a stronger stress-adaptive signature, including downregulation of genes in acyl-CoA biosynthesis (*tecrb*,* hacd2*,* acsl2*) and the TCA cycle (*idh2*,* aco2*), alongside strong upregulation of cytokine networks (*il1β*,* cxcl18b*). This coincided with KEGG pathway enrichment for fatty acid elongation, steroid biosynthesis, and PPAR signaling. In contrast, fed fish maintained hepatic architecture and showed a more restrained immune response, with enriched oxidoreductase activity in sterol metabolism (*dhcr24*,* dhcr7*,* tm7sf2*), though these anabolic pathways were also suppressed. Fin-clipping was associated with greater pro-inflammatory signaling, but these patterns should be interpreted in the context of the relevant sham control structure.

**Conclusion:**

Our findings suggest that feeding status shapes the early hepatic response during septicemia. Short-term fasting appears to prime the liver for a catabolic, stress-responsive state that may compromise metabolic resilience, while feeding supports homeostasis when compared to fed clipped or unclipped controls. This provides a rationale for nutritional management as an important strategy to enhance metabolic readiness and disease resilience in aquaculture, offering novel targets for functional genomics studies in fish health.

**Supplementary Information:**

The online version contains supplementary material available at 10.1186/s12864-026-12988-1.

## Introduction

Infectious disease represents a persistent and economically devastating challenge in intensive aquaculture [1, 2]. Outbreaks rarely occur in isolation but are instead mediated by a confluence of husbandry and environmental stressors, including variable feeding schedules and incidental physical injury during handling [3, 4]. These concurrent stressors likely engage complex host response pathways, yet the systems-level mechanisms by which they interact to influence disease progression and outcomes remain poorly resolved. Therefore, understanding these interactions requires using experimental designs that capture the multifactorial reality of production systems rather than single-factor models.

The liver is a vital metabolic and immunological organ that coordinates physiological responses in an animal host [5]. It orchestrates energy homeostasis, biosynthesis, detoxification, and systemic immune responses [5–8]. During infection, the liver undergoes profound metabolic reprogramming to fuel immune defense, a process termed immunometabolism [9]. The nature of this reprogramming is likely shaped by the host’s pre-infection physiological state [10]. However, the precise transcriptional profile of these early hepatic immunometabolic shifts in channel catfish, especially under compounded stress, is not well-defined.

In channel catfish (*Ictalurus punctatus*), a species of considerable aquacultural importance, infections such as those caused by virulent *Aeromonas hydrophila* (vAh) present a significant challenge to both innate immunity and metabolic resilience. Virulent *A. hydrophila* is one cause of motile *Aeromonas* septicemia (MAS), a rapidly progressing disease characterized by hemorrhagic lesions, hepatic dysfunction, and high mortality [11–16]. Epithelial barriers, such as the skin and fins, are primary ports of entry for vAh, and physical injury is a known exacerbating factor [17, 18]. While the individual impacts of fasting or injury on infection susceptibility have been documented [17, 18], their combined effect on the host’s central immunometabolic organ at a genome-wide transcriptional level has not been investigated.

Current research has provided insights into pathological progression of vAh infection [13, 14]. However, a significant knowledge gap exists in our understanding of how pre-infection cues (fed vs. fasted status) and concurrent physical insult (fin-clipping) interact to reshape the hepatic transcriptome during the critical early hours of infection. Such an integrated analysis is necessary for mechanistic prediction of host resilience during acute infectious diseases like MAS.

We used a MAS disease model to evaluate feeding status (fed or fasted) with or without adipose fin‑clipping infection groups and uninfected controls (fed with or without fin-clipping) through histological and transcriptomic (RNA-Seq) analysis to investigate the hepatic response to vAh in channel catfish. We hypothesized that feeding status would be the primary factor driving immunometabolic reprogramming in the liver, and physical injury would further modulate this response by amplifying specific inflammatory pathways. To test this hypothesis, we compared fed versus fasted and fin-clipped versus unclipped fish across early timepoints (2, 4, and 8 h post-challenge). Specifically, our objectives were to: (1) identify the unique and shared transcriptional signatures associated with each stressor, (2) characterize the resulting immunometabolic pathway alterations, and (3) provide a molecular framework linking MAS feed status and injury to hepatic resilience.

## Materials and methods

### Ethics statement

All fish experiments were conducted at the USDA-ARS Aquatic Animal Health Research Unit (AAHRU) under an approved AAHRU Institutional Animal Care and Use Committee protocol number 064 approved on June 15, 2023, and conformed to USDA-ARS Policies and Procedures 130.4.v5.

### Experimental animals and husbandry

Briefly, Delta Select channel catfish (average weight 35 ± 2 g, *n* = 736) were acclimated for 14 days in recirculating aquaculture system (RAS) with daily monitoring of temperature (28 ± 2 °C), pH (7.5 ± 0.5), and dissolved oxygen (7.0 ± 2.0 mg/L). Prior to experimentation, sampled fish (*n* = 5) were screened and confirmed negative for *Aeromonas hydrophila*, indicating the absence of detectable baseline infection in the study population. Aeration was supplied using air stones, and a 12 h:12 h light: darkness photoperiod was used. During acclimation, the channel catfish were fed the same pre-challenge commercial 32% protein 4.8 mm catfish pellets at 3% of their average body weight. The proximate composition of the diet included crude protein 32%, crude fat 5%, and crude fiber 4%.

### Bacterial culture and challenge stock preparation

The vAh isolate ALG-15-097 previously characterized and associated with MAS outbreaks [16], was used for challenge. Briefly, a single colony from tryptic soy agar supplemented with 5% sheep blood (Remel, Lenexa, KS) was inoculated in tryptic soy broth (TSB) (Becton, Dickinson and Co., Sparks, MD) with 0.4 mM deferoxamine mesylate (DFO) (Sigma-Aldrich, St. Louis, MO) and incubated at 28 ± 2 °C with 115 rpm constant shaking for 24 h. Bacterial concentration in colony-forming units (CFU) was determined by measuring the optical density (OD) at 540 nm using an Ultrospec 2100 pro UV/Visible Spectrophotometer (Pharmacia) and confirmed by serial dilution plating on TSA-blood plate. This concentration was 1.5 × 10^9^ CFU/mL. The control inoculum consisted of sterile TSB + DFO prepared identically but without bacterial inoculation.

### Experimental design and challenge procedure

The MAS infected groups were FCF, fin-clipped fed; FCN, fin-clipped fasted, NCF, unclipped fed; NCN, unclipped fasted. The sham control groups were designed to reflect baseline husbandry conditions (cNCF, unclipped fed), and the MAS model (cFCF, fin-clipped fed). Both groups were sham-infected with sterile TSB. On the day of the challenge, channel catfish in the fed groups were offered feed at 3% of their body weight 2 h prior to challenge. The fasted groups received their last feeding the day before the challenge, approximately 24 h prior to the challenge. For the fin-clipped procedure, channel catfish were anesthetized with 100 mg/L of Syncaine (MS-222) (Syndel, Ferndale, WA) buffered with sodium bicarbonate and observed for the absence of opercular movement after 5 min to confirm full sedation. The adipose fin (Af) was clipped at its base using sterile scissors as previously described [17]. The unclipped groups were anesthetized and handled identically but without the clipping procedure. Following treatment assignment, fish were randomly allocated into one of four replicate tanks per treatment group (*n* = 25 fish per tank; total *n* = 100 fish per treatment). Each replicate tank contained 57 L of aerated water. Challenge was performed by static immersion in 10 L, as follows. Sterile broth (sham challenge) was added to the appropriate control tanks (100 mL media) while the bacterial suspension was added to each tank (10 L media + 100 mL bacteria) to achieve the final concentration of 1.5 × 10⁷ CFU/mL. Fish were exposed for 1 h, after which the water flow was resumed at 0.5 L/min for the duration of the challenge. The sham control groups were designed to reflect baseline husbandry conditions in which fish are normally fed, while incidental injury may or may not occur during feeding/handling; accordingly, both sham groups were fed, with one group fin-clipped and the other unclipped. Mortality was monitored for 72 h since no mortality were recorded after this timepoint; survival data from this challenge has been previously published [18].

### Tissue sampling for histopathology and transcriptomics

At 2, 4, and 8 h post-challenge (hpc), six fish per treatment group per timepoint (total *n* = 72) were randomly sampled and euthanized using an overdose of buffered Syncaine (> 300 mg/L). A ventral midline incision was performed to expose the visceral cavity. The liver was carefully dissected and immediately fixed in 10% neutral buffered formalin for 48 h. The channel catfish in the control groups were sampled at 2 hpc only, following the same protocol (total *n* = 12). For transcriptomics, another group of six fish per treatment group per timepoint (total = 72) were randomly sampled and euthanized using an overdose of buffered Syncaine (> 300 mg/L). After a ventral midline incision was performed to expose the visceral cavity, liver tissue (20–30 mg) was placed into 1 mL of RNA stabilization solution (RNAlater, ThermoFisher Scientific, Waltham, MA) and stored at -80 °C until needed. Similarly, the channel catfish in the control groups were sampled at 2 hpc only, following the same protocol (total *n* = 12).

### Histopathology processing and analysis

The formalin-fixed liver samples were processed routinely after they were placed in cassettes as previously reported [18]. Briefly, the samples were dehydrated through a graded ethanol series, cleared in xylene, and embedded in paraffin wax. Thin sections, 5 μm thick, were then cut from the embedded tissues and stained with hematoxylin and eosin (H&E). The stained sections on glass slides were examined under a light microscope (Olympus BX41, Olympus America, Bartlett, TN) at total magnification of between 40-400x.

### RNA extraction, sequencing and bioinformatics

Total RNA liver samples (*n* = 84) were assessed for quality and quantity using the 4200 TapeStation System with the RNA ScreenTape assay (Agilent Technologies, Santa Clara, CA) and a BioTek Cytation 1 Plate Reader with the BioTek Take3 Microvolume Plate (Agilent Technologies, Santa Clara, CA). Total RNA liver samples (*n* = 84) meeting the quality metrics were shipped to a service provider (SeqMatic, Fremont, CA) for RNA sequencing on an Illumina NovaSeq X Instrument (San Diego, CA) in a 2 × 150 bp paired-end configuration with a target sequencing depth of > 25 M paired-end reads/sample. The metrics included RNA concentration greater than 20 ng/µL, A260:A280 between 1.8 and 2.0 and RIN score above 7.0 (Table S1). Sequencing libraries were made with the Illumina Stranded mRNA Ligation Prep Kit (Illumina, San Diego, CA) according to the manufacturer’s protocol.

Raw, demultiplexed reads, with a minimum of 25 M paired-end reads/sample were provided for analysis. Bioinformatics processing was carried out using OmicsBox software [19] and R-Bioconductor packages. The initial preprocessing of the raw FASTQ files involved quality control using FASTQC [20] and Trimmomatic [21] with default settings to remove low-quality bases, short reads, and Illumina adapter sequences. The channel catfish genome (Coco_2.0 assembly; GenBank accession GCA_001660625.3) was used for alignment. Quality-controlled (QC) reads were aligned to the genome using the STAR aligner [22] with 2-pass mapping and an overhang length of 149. The resulting BAM files were evaluated for alignment quality using RSeQC [23–25], generating metrics such as transcript integrity numbers (TINs). Gene-level counts were obtained from the QC-checked BAM files using HTSeq [26], with settings for strand-specific orientation, exon-based quantification, and union mode for overlapping reads. Differential expression analysis was conducted with edgeR [27]. First, genes with low counts were filtered via the ‘filterByExpr’ function using the default parameters (min.count = 10; min.total.count = 15). Samples were then normalized using the Trimmed Mean of M values (TMM) method. The Quasi Likelihood F-test with the additional parameter of robust=true was used for pairwise comparisons between infected groups and sham controls, as such, these comparisons were interpreted as infection responses relative to the fed baseline conditions. Genes were classified as differentially expressed (DEGs) if they had an adjusted p-value below 0.05 and exhibited more than a 2-fold up- or downregulation compared to the control (*P*-adj < 0.05, FC > ± 2).

### Functional enrichment and categorization analysis

Gene Ontology (GO) enrichment analysis was conducted on the DEG lists to interpret their biological relevance across three GO domains: Biological Process (BP), Cellular Component (CC), and Molecular Function (MF). All functional annotations and enrichment tests were carried out using the OmicsBox platform v3.4.6 [19]. First, the channel catfish RefSeq transcriptome, derived from the Coco_2.0 genome assembly, was imported into OmicsBox. Transcripts were functionally annotated using BLASTx searches (E-value threshold < 1 × 10⁻³), InterPro protein domain analysis, and assignment of Enzyme Commission (EC) numbers where available. GO terms were mapped to each transcript, generating a comprehensive, functionally annotated reference transcriptome [28]. For each DEG list, over-representation of GO terms was assessed via Fisher’s exact test. The entire annotated transcriptome served as the reference set, and the DEG list from each comparison constituted the test set. Terms with a false discovery rate (FDR) < 0.05 were considered significantly enriched. To complement the discrete DEG-based approach, a Gene Set Enrichment Analyses (GSEA) [29] was also performed within OmicsBox using the default settings. Briefly, gene lists from each pairwise comparison were ranked and submitted for GSEA with 1,000 permutations to generate significance (FDR < 0.05).

Each significant DEG list was functionally profiled by compiling annotations from GO, EC numbers, InterPro domains, and KEGG pathway assignments as provided by the OmicsBox Combined Pathway Analysis pipeline. This integrated output allowed a multi-faceted interpretation of the biological themes associated with the transcriptional changes in each comparison. To further contextualize the functional data, annotated sequences were also assigned to corresponding biological pathways using the Kyoto Encyclopedia of Genes and Genomes (KEGG) database within the same software environment.

Finally, to broadly classify DEGs into major functional themes as relevant to liver physiology, a manually curated list of keywords related to ‘metabolism’ and ‘immune surveillance’ were established (Table S2). A custom R script utilizing stringr package (v1.5.1) scanned the gene description field for each DEG against these keyword lists. Genes were assigned to one or both categories based on keyword matches. This categorized data was then used to generate the alluvial diagram to visualize the flow of DEGs across treatments, timepoints, and functional categories using the ggalluvial package (v0.12.5). For heatmap visualizations, selected DEGs were ranked by absolute log2 fold-change, reshaped into a gene-by-treatment matrix, and hierarchical clustering was performed on the resulting log2 fold-change matrix for both genes and treatment groups.

### RT-qPCR validation

Select genes were independently assessed via reverse transcription quantitative polymerase chain reaction (RT-qPCR) to validate the RNA sequencing analyses. An aliquot of liver total RNA liver (*n* = 36) was used for RNA-Seq gene validation. For each liver sample, the RNA was normalized to deliver 200 ng RNA into a 20 µL reverse transcription (RT) reaction. cDNA synthesis was performed using the LunaScript^®^ RT SuperMix Kit (New England Biolabs, Ipswich, MA). Reactions contained 4 µL of LunaScript RT SuperMix (5X) and 200 ng of template RNA, and the volume was adjusted to 20 µL with nuclease-free water. As a control, to rule out the presence of DNA in the samples, no-RT reactions were prepared for each of the samples, along with no-template controls (negative control). Reaction conditions for cDNA synthesis included primer annealing at 25 °C for 2 min, cDNA synthesis at 55 °C for 10 min and heat inactivation at 95 °C for 1 min.

RT-qPCR assays were performed using the Roche LightCycler 96 (Roche Diagnostics, Indianapolis, IN). The genes selected for validation were *il-1βa*,* tlr5*,* tecrb*,* dhcr7*,* acsl2*, and *hacd2*. Gene expression data was normalized using *18 S* rRNA as the reference gene. Primer sequences, expected amplicon sizes and gene descriptions are provided in Table S6. All RT-qPCR reactions were carried out in triplicate under the following conditions: 95 °C for 15 s, followed by 45 cycles at 95 °C for 15 s, 60 °C for 30 s, followed by melting curve analysis. Primer efficiency of 100% was assumed for all targets and reference gene. Cycle threshold (Ct) values were collected, and fold-change between each comparison was determined using the 2^−ΔΔCT^ method [30]. *P*-values were calculated using a Student’s t-test.

### Data availability

The RNA sequencing datasets generated for this study have been deposited to the NCBI Gene Expression Omnibus (GEO) repository and can be found under accession number GSE280610. All other data that support the findings of this study have been included in the manuscript and Supplementary Materials.

## Results

### Survival and gross pathology confirm the synergistic impact of injury and fasting

The Kaplan-Meier survival curve [21] demonstrated that the highest mortality was observed in fasted, fin-clipped fish (FCN, 77%), followed by fed, fin-clipped (FCF, 70%) and fed, unclipped (NCF, 62%) groups, with fasted, unclipped (NCN, 45%) fish showing the highest survival (*p* < 0.05). This established a clear mortality pattern where fin-clipping exacerbated outcomes, and fasted status further increased susceptibility, particularly in injured fish. External signs of motile *Aeromonas* septicemia (MAS), including exophthalmia, fin reddening, and petechial hemorrhages, were evident across all infected groups as early as 2 hpc.

### Histopathological alterations are primarily evident in fasted, uninjured fish

**Fig. 1:**
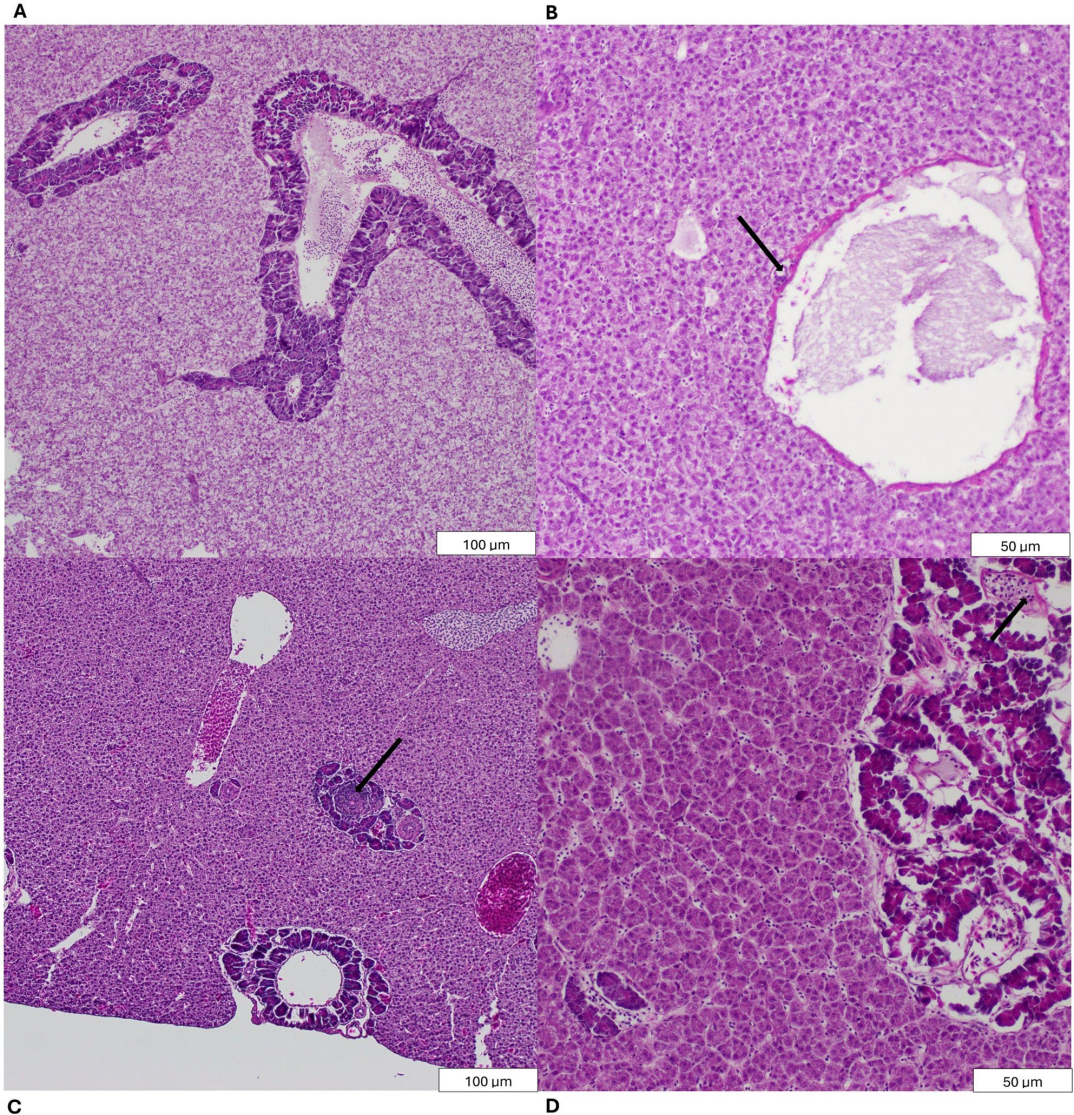
Photomicrographs of channel catfish liver samples. **A** The normal morphology of the liver showing normal hepatocytes and blood vessels, 100x, H&E, NCN, control. **B** Moderate vascular congestion and edema (arrow), 200x, H&E, NCN, at 2 HPC. **C** Moderate vascular congestion, edema, and lymphoplasmacytic infiltration (arrow), 200x, H&E, NCN, at 4 HPC. **D** Moderate vascular congestion, and moderate to severe edema, 200x, H&E, NCN, at 8 HPC

Histological evaluation of liver tissue from control fish groups revealed a well-organized hepatic architecture with clearly defined hepatopancreatic structures, maintaining the integrity of the hepatic cords, sinusoidal spaces, central veins and pancreatic acini, without evidence of cellular degeneration, inflammatory infiltration, or vascular disruption (Fig. 1A). Among treatment groups, NCN group exhibited moderate vascular congestion and edema at 2 hpc (Fig. 1B). By 4 hpc, these features were accompanied by noticeable lymphoplasmacytic infiltration within the hepatic parenchyma (Fig. 1C) and at 8 hpc, moderate to severe edema and pronounced vascular congestion were dominant (Fig. 1D). In contrast, the other three treatment groups (FCF, NCF, FCN) showed minimal to no detectable histopathological changes across the same timepoints, with preserved tissue architecture (Figure S1).

### The hepatic transcriptomic landscape is more associated with feeding status than injury status

**Fig. 2:**
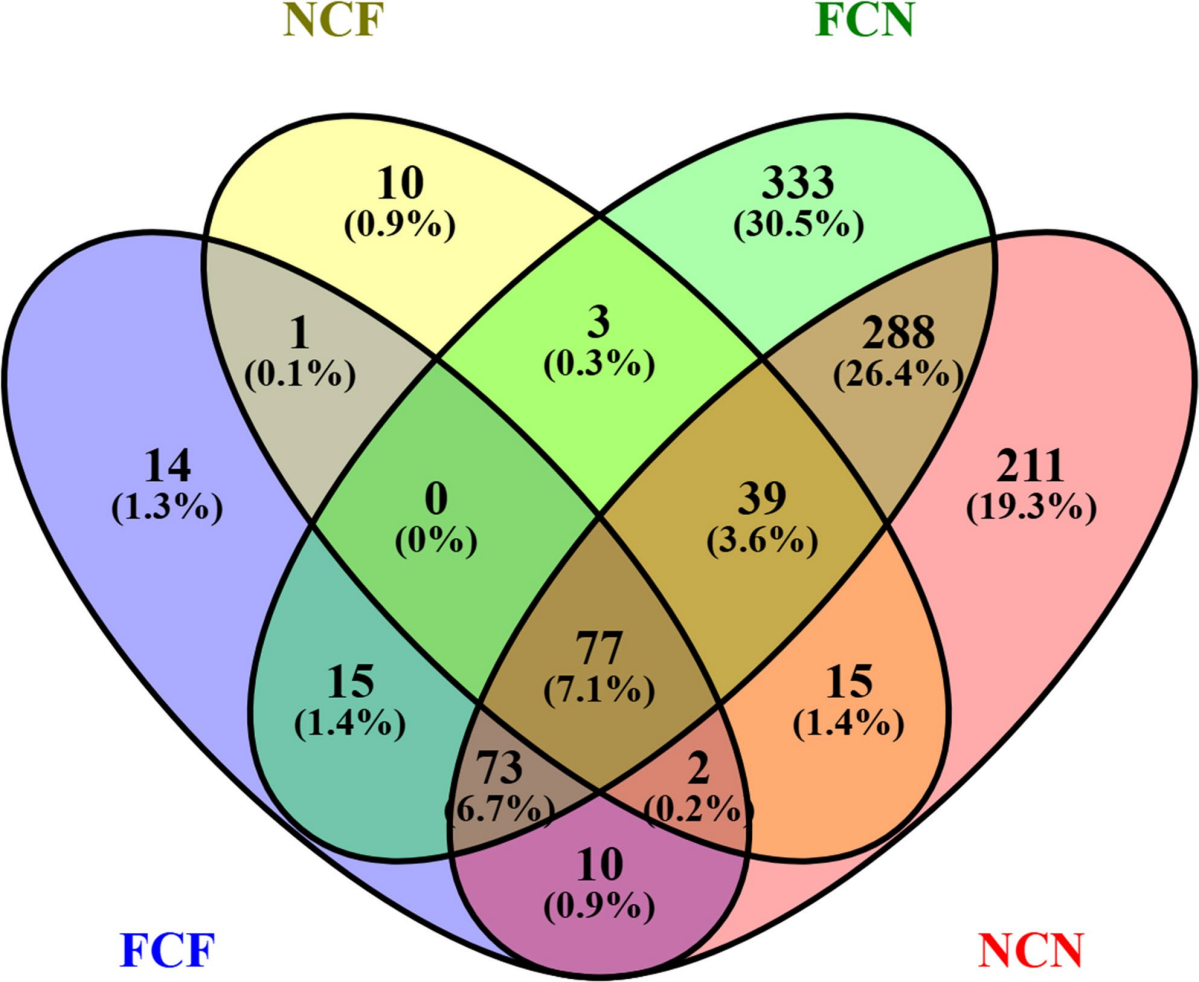
Venn diagram displaying unique and commonly expressed DEGs at 2, 4, 8 HPC in the different treatment groups of the liver (*P* < 0.05). Groups: Fin clip fed (FCF), No fin clip fed (NCF), Fin clip fasted (FCN), No fin clip fasted (NCN)

To delineate the pre-mortality transcriptional response, we focused on genes that were differentially expressed in each treatment group compared to the control groups and then identified the conserved DEGs across all three timepoints. The DEGs were interpreted relative to the two sham groups as baseline conditions, and direct comparisons among infected groups were used to assess feeding- and injury-associated response patterns. The distribution of these “conserved DEGs” is shown in Fig. 2, Table S3 (*n* = 77). The fasted groups harbored most of the unique transcriptional changes: 333 genes were unique to FCN and 211 to NCN. In contrast, fed groups showed minimal unique responses (FCF: 14 genes; NCF: 10 genes). A substantial overlap of 288 genes was shared between the two fasted groups (FCN & NCN), while the fed groups shared only one gene. This pattern suggests that feeding status (fed vs. fasted) was more associated than injury status with the hepatic transcriptomic response to vAh challenge under the present experimental and control conditions.

### Consistent DEGs are enriched for immunometabolic functions

**Fig. 3:**
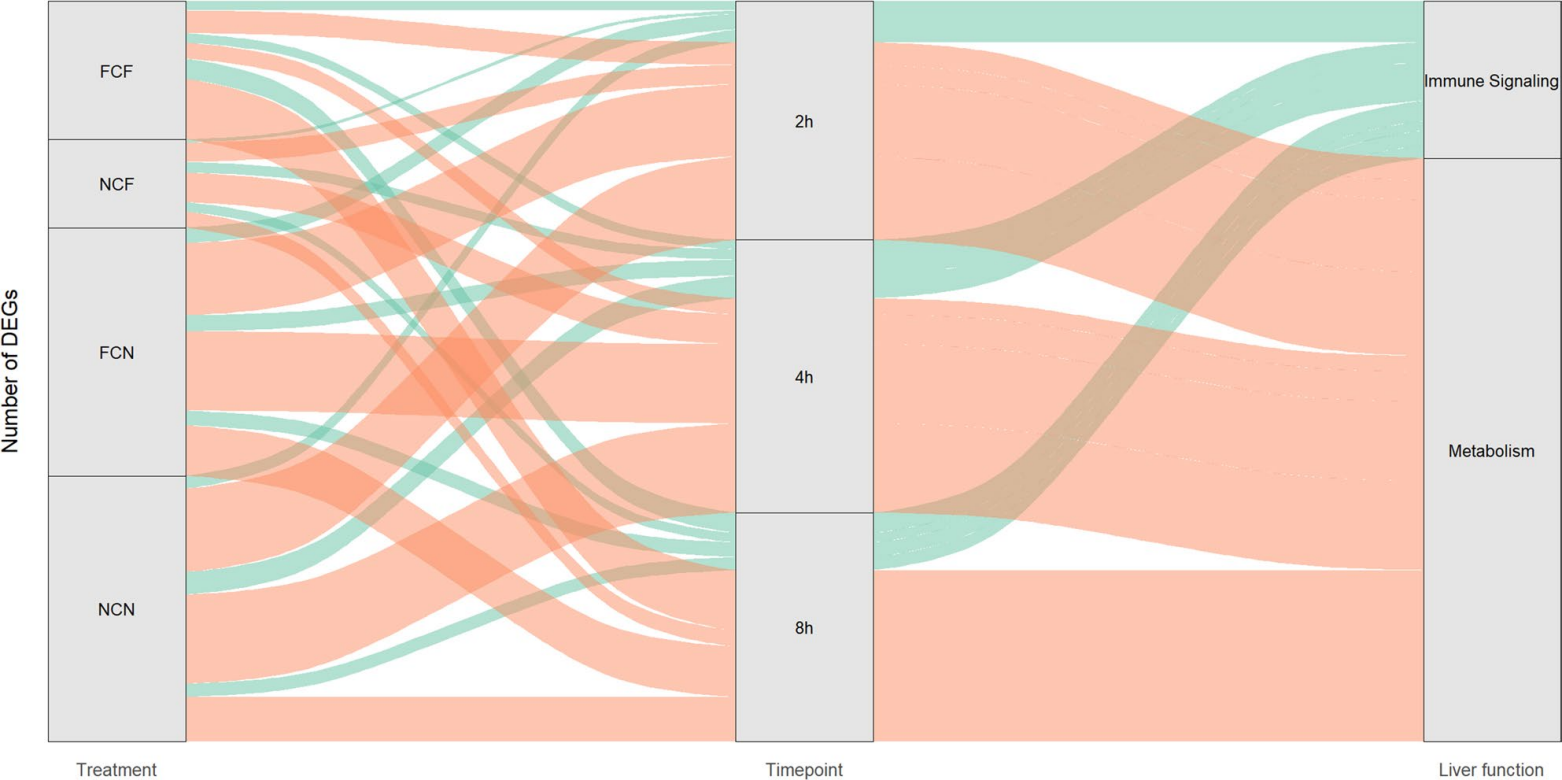
Alluvial plot of immune signaling and metabolism related DEGs within the liver of channel catfish at 2, 4, and 8 hpc in each treatment group. Most DEGs were metabolism-related (~ 80%), the remaining were immune-related. Groups: Fin clip fed (FCF), No fin clip fed (NCF), Fin clip fasted (FCN), No fin clip fasted (NCN)

Next, the categorization of the DEGs based on functional annotations revealed that 78.8% were associated with metabolic processes (carbohydrate, lipid, protein metabolism), while 21.2% were linked to immune signaling as depicted for each treatment group (Fig. 3, Table S2). This predominance of metabolism-associated transcripts underscores the liver’s central role in metabolic reprogramming during infection. Representative immune-related DEGs included canonical pro-inflammatory cytokines and receptors such as interleukin 1 beta (*il1b*), tumor necrosis factor alpha-induced protein 3 (t*nfaip3*), chemokine ligand 19a (*ccl19a.1*), and interleukin-6 receptor (*il6r*), Key metabolic DEGs spanned pathways from glycolysis(hexokinase (*hk*) and pyruvate kinase (*pk*) to fatty acid synthesis and oxidation (fatty acid synthase (*fasn*) and carnitine palmitoyltransferase (*cpt1a*), and protein metabolism (proteasome subunits and transaminases).

### Gene ontology analysis reveals pathway enrichment patterns with feeding status

**Fig. 4:**
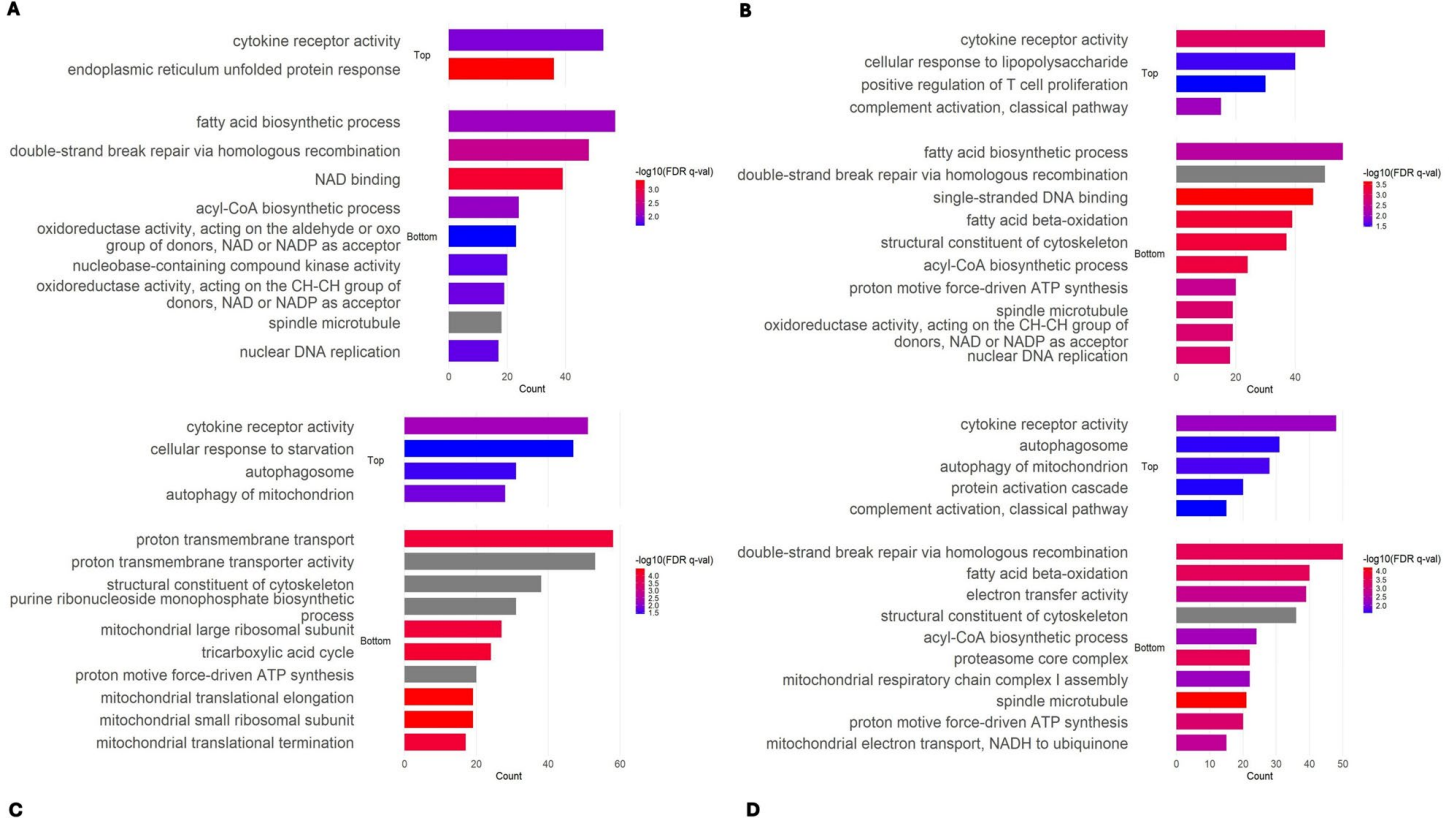
Horizontal bar plots showing enriched Gene Ontology (GO) terms associated with differentially expressed genes (DEGs) in the fed groups, (**A**) FCF and (**B**) NCF, and in the fasted groups, (**C**) FCN and (**D**) NCN, after challenge with virulent *Aeromonas hydrophila*. In each panel, the upper section represents GO terms enriched among upregulated DEGs, whereas the lower section represents GO terms enriched among downregulated DEGs. The x-axis shows the number of DEGs mapped to each GO term, and the y-axis shows the enriched GO terms. Bar color indicates false discovery rate (FDR < 0.05)

GO enrichment analysis performed on the conserved DEGs from each group illuminated distinct biological themes (Fig. 4, Table S4). First, in the fed groups (FCF and NCF), enriched terms were related to specific immune-regulatory functions, such as cytokine receptor activity (GO:0004896) in FCF (Fig. 4A, Table S4) and complement activation (GO:0006958), cellular response to lipopolysaccharide (GO:0071222), and positive regulation of T cell proliferation (GO:0042102) in NCF (Fig. 4B, Table S4). Notably, both fed groups showed significant negative enrichment (i.e., coordinated downregulation) of pathways central to mitochondrial energy metabolism and lipid biosynthesis, including fatty acid biosynthetic process (GO:0006633) and acyl-CoA biosynthetic process (GO:0071616).

Secondly, the fasted groups (FCN and NCN) displayed a transcriptional profile characteristic of a catabolic, resource-scarce state stress-response signature. Positive enrichment included terms autophagosome (GO:0005776), autophagy of mitochondrion (GO:0000422), cytokine receptor activity (GO:0004896) (Fig. 4C-D, Table S3). Mirroring the fed groups, but with a greater magnitude, fasted groups also exhibited strong negative enrichment of mitochondrial metabolic processes and lipid biosynthesis pathways including fatty acid biosynthesis (GO:0006633), fatty acid beta oxidation (GO:0006635), and acyl-CoA biosynthesis (GO:0071616).

### Feeding status is associated with immune and metabolic transcriptional signatures

**Fig. 5:**
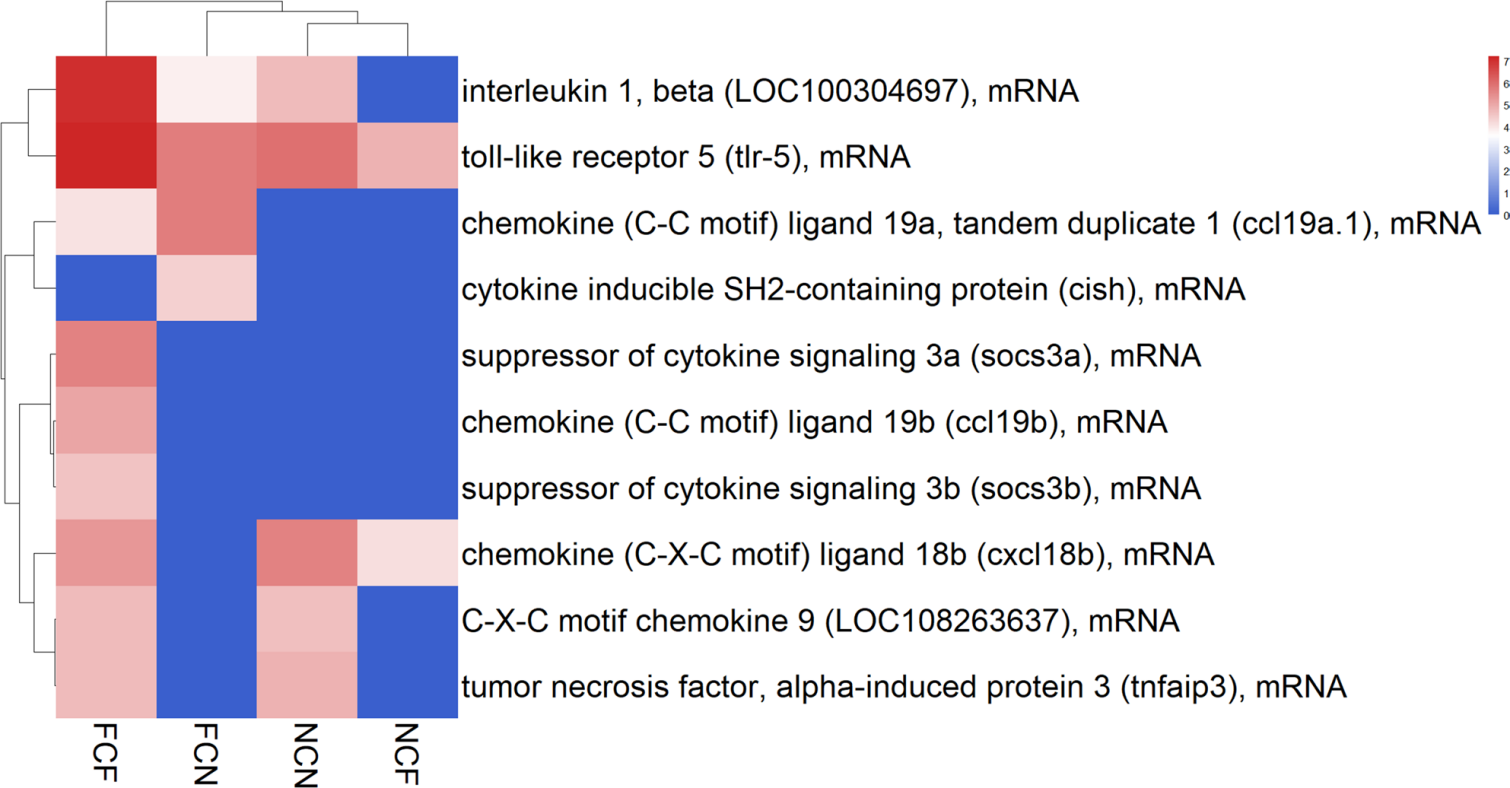
Heatmap of selected upregulated differentially expressed genes (DEGs) associated with immune and inflammatory responses in channel catfish following challenge with virulent *Aeromonas hydrophila*. Genes were selected from the ranked DEG list based on absolute log2 fold-change, and the heatmap displays the subset included in the final figure. Rows represent genes and columns represent treatment groups. Cell color indicates log2 fold-change relative to the corresponding control comparison. Hierarchical clustering of rows and columns was performed on the log2 fold-change matrix to group genes and treatment groups with similar expression-response patterns

**Fig. 6:**
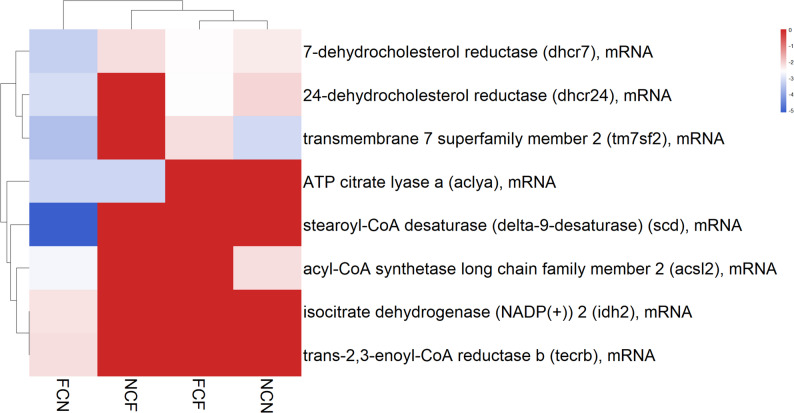
Heatmap of selected downregulated differentially expressed genes (DEGs) associated with lipid and sterol metabolic processes in channel catfish following challenge with virulent *Aeromonas hydrophila*. Genes were selected from the ranked DEG list based on absolute log2 fold-change, and the heatmap displays the subset included in the final figure. Rows represent genes and columns represent treatment groups. Cell color indicates log2 fold-change relative to the corresponding control comparison. Hierarchical clustering of rows and columns was performed on the log2 fold-change matrix to group genes and treatment groups with similar response patterns.

Further delving into the specific enriched GO terms revealed how feeding status shapes immunometabolic response. For immune signaling, the cytokine receptor activity (GO:0004896) was enriched across all groups but was most extensive in fasted fish (32 transcripts in FCN vs. 16 in FCF). These included interleukin-6 cytokine-family signal-transducer (*il6st*), interleukin-1 receptor-like 2 (*il1rl2*) and interleukin-10 receptor subunit beta (*il10rβ*) (Fig. 5; Table 1). While toll-like receptor 5 (*tlr5*) and *il6st* were upregulated in all infected groups, fasted fish uniquely exhibited significant upregulation of potent pro-inflammatory mediators as described above. For lipid and sterol metabolism, the acyl-CoA biosynthetic process (GO:0071616) was negatively enriched in all groups but involved more core genes in fasted fish [2,3-enoyl-CoA reductase b (*tecrb*), 3-hydroxyacyl-CoA dehydratase 2 (*hacd2*), and acyl-CoA synthetase long-chain family member 2 (*acsl2*)], all of which were downregulated (Fig. 6; Table 2). Conversely, the oxidoreductase activity acting on CH-CH group donors (GO:0016628), heavily populated by cholesterol biosynthesis genes [24-dehydrocholesterol reductase (*dhcr24*), 7-dehydrocholesterol reductase (*dhcr7*), and transmembrane 7 superfamily member 2 (*tm7sf2*)] were uniquely and significantly negatively enriched in the fed groups, though these transcripts were also downregulated.

eatmap displays the subset included in the final figure. Rows represent genes and columns represent treatment groups. Cell color indic

**Table 1:** Selected DEGs assigned to immune function gene ontology identified among treatment DEGs shared at the (2-, 4- and 8-h) time intervals in the catfish liver tissue post vAh challenge. DEGs; up- or down-regulated related to a proinflammatory response or pathogen recognition (FDR < 0.05).

Trt	Feature	F	FC	FDR	Description
FCF	NM_001200229.1	195.44	148.83	0.00	toll-like receptor 5 (tlr-5)
NCF	NM_001200229.1	26.53	28.95	0.01	toll-like receptor 5 (tlr-5)
FCN	NM_001200229.1	22.69	54.10	0.01	toll-like receptor 5 (tlr-5)
NCN	NM_001200229.1	46.27	60.99	0.00	toll-like receptor 5 (tlr-5)
FCF	NM_001200202.1	46.52	40.46	0.00	chemokine (C-X-C motif) ligand 18b (cxcl18b)
FCF	NM_001200220.1	57.07	133.02	0.00	interleukin 1, beta (LOC100304697)
FCF	NM_001201254.1	46.43	50.65	0.00	suppressor of cytokine signaling 3a (socs3a)
FCF	XM_017451519.3	64.26	17.06	0.00	chemokine (C-C motif) ligand 19a, tandem duplicate 1 (ccl19a.1)
FCF	XM_017464663.3	24.24	26.41	0.00	C-X-C motif chemokine 9 (LOC108263637)
FCF	XM_017464718.3	36.66	26.68	0.00	tumor necrosis factor, alpha-induced protein 3 (tnfaip3)
FCF	XM_017475092.2	17.94	-8.17	0.01	transforming growth factor, beta-induced (tgfbi)
FCF	XM_017480048.3	34.62	9.58	0.00	cytokine inducible SH2-containing protein (cish)
FCF	XM_017483973.3	74.83	24.25	0.00	suppressor of cytokine signaling 3b (socs3b)
FCF	XM_017488873.3	87.88	10.68	0.00	suppressor of cytokine signaling 1a (socs1a)
FCF	XM_017489233.3	95.66	4.52	0.00	interleukin 6 cytokine family signal transduce (il6st)
FCF	XM_017489912.3	35.04	6.88	0.00	nuclear factor, interleukin 3 regulated (nfil3)
FCF	XM_017494200.2	49.22	7.73	0.00	interleukin-1 receptor-associated kinase 3 (irak3)
FCF	XM_053687071.1	205.40	33.82	0.00	chemokine (C-C motif) ligand 19b (ccl19b)
NCF	NM_001200202.1	36.31	17.72	0.00	chemokine (C-X-C motif) ligand 18b (cxcl18b)
NCF	XM_017464718.3	28.64	8.47	0.01	tumor necrosis factor, alpha-induced protein 3 (tnfaip3)
NCF	XM_017489233.3	38.47	4.18	0.00	interleukin 6 cytokine family signal transduce (il6st)
FCN	NM_001200202.1	22.46	9.36	0.01	chemokine (C-X-C motif) ligand 18b (cxcl18b)
FCN	NM_001200220.1	23.86	14.43	0.01	interleukin 1, beta (LOC100304697)
FCN	NM_001201254.1	23.65	12.57	0.01	suppressor of cytokine signaling 3a (socs3a)
FCN	XM_017451519.3	26.22	52.72	0.00	chemokine (C-C motif) ligand 19a, tandem duplicate 1 (ccl19a.1)
FCN	XM_017455297.3	18.62	-2.12	0.01	interleukin enhancer binding factor 2 (ilf2)
FCN	XM_017464663.3	13.14	6.75	0.02	C-X-C motif chemokine 9 (LOC108263637)
FCN	XM_017464718.3	27.54	7.80	0.00	tumor necrosis factor, alpha-induced protein 3 (tnfaip3)
FCN	XM_017468925.3	16.48	2.38	0.01	interleukin 6 receptor (il6r)
FCN	XM_017470457.3	34.15	3.32	0.00	interleukin-1 receptor-like 2 (zmp:0000000936)
FCN	XM_017470626.3	20.33	2.80	0.01	interleukin-10 receptor subunit beta (LOC108266840)
FCN	XM_017475092.2	10.07	-4.65	0.04	transforming growth factor, beta-induced (tgfbi)
FCN	XM_017480048.3	18.29	20.83	0.01	cytokine inducible SH2-containing protein (cish)
FCN	XM_017483973.3	18.04	9.13	0.01	suppressor of cytokine signaling 3b (socs3b)
FCN	XM_017488873.3	20.78	9.75	0.01	suppressor of cytokine signaling 1a (socs1a)
FCN	XM_017489233.3	19.06	5.16	0.01	interleukin 6 cytokine family signal transduce (il6st)
FCN	XM_017489912.3	26.52	3.84	0.00	nuclear factor, interleukin 3 regulated (nfil3)
FCN	XM_017493717.3	17.98	3.32	0.01	nuclear factor, interleukin 3 regulated, member 6 (nfil3-6)
FCN	XM_017494200.2	28.48	4.07	0.00	interleukin-1 receptor-associated kinase 3 (irak3)
NCN	NM_001200202.1	40.89	51.87	0.00	chemokine (C-X-C motif) ligand 18b (cxcl18b)
NCN	NM_001200220.1	36.90	26.43	0.00	interleukin 1, beta (LOC100304697)
NCN	NM_001201254.1	24.49	11.70	0.00	suppressor of cytokine signaling 3a (socs3a)
NCN	XM_017459704.3	16.89	2.24	0.01	tumor necrosis factor (ligand) superfamily, member 10 like 4 (tnfsf10l4)
NCN	XM_017464663.3	40.73	25.01	0.00	C-X-C motif chemokine 9 (LOC108263637)
NCN	XM_017464718.3	40.02	28.84	0.00	tumor necrosis factor, alpha-induced protein 3 (tnfaip3)
NCN	XM_017470457.3	17.98	2.60	0.01	interleukin-1 receptor-like 2 (zmp:0000000936)
NCN	XM_017470626.3	23.14	2.57	0.00	interleukin-10 receptor subunit beta (LOC108266840)
NCN	XM_017480048.3	12.43	9.86	0.02	cytokine inducible SH2-containing protein (cish)
NCN	XM_017483973.3	33.54	12.63	0.00	suppressor of cytokine signaling 3b (socs3b)
NCN	XM_017489233.3	48.85	5.80	0.00	interleukin 6 cytokine family signal transduce (il6st)
NCN	XM_017494200.2	38.19	10.74	0.00	interleukin-1 receptor-associated kinase 3 (irak3)

**Table 2:** Selected DEGs related to lipid metabolic function identified among treatment DEGs shared at the (2-, 4- and 8-h) time intervals in the catfish liver tissue post vAh challenge. DEGs; up- or down-regulated related to Acyl-CoA biosynthesis and lipid or sterol processes (FDR < 0.05).

Trt	Feature	F	FC	FDR	Description
FCF	XM_017456076.3	20.34	-6.08	0.01	24-dehydrocholesterol reductase (dhcr24)
FCF	XM_017478833.3	30.98	-5.76	0.00	7-dehydrocholesterol reductase (dhcr7)
FCF	XM_017488985.3	17.23	-4.59	0.01	transmembrane 7 superfamily member 2 (tm7sf2)
FCN	XM_017453626.3	10.90	-2.87	0.03	2’,3’-cyclic nucleotide 3’ phosphodiesterase (cnp), transcript variant X1
FCN	XM_017457211.3	85.91	-9.37	0.00	ATP citrate lyase a (aclya)
FCN	XM_017465598.2	15.04	-4.65	0.02	isocitrate dehydrogenase (NADP(+)) 2 (idh2)
FCN	XM_017487208.3	9.60	-2.14	0.04	isocitrate dehydrogenase [NAD] subunit gamma, mitochondrial (LOC108275983)
FCN	XM_053677583.1	20.50	-2.02	0.01	succinate-CoA ligase GDP/ADP-forming subunit alph (suclg1)
FCN	XM_053685363.1	9.54	-3.99	0.05	aconitase 2, mitochondrial (aco2)
FCN	NM_001201135.1	20.26	-4.48	0.01	trans-2,3-enoyl-CoA reductase b (tecrb)
FCN	XM_017456076.3	23.28	-8.55	0.01	24-dehydrocholesterol reductase (dhcr24)
FCN	XM_017478833.3	36.60	-9.97	0.00	7-dehydrocholesterol reductase (dhcr7)
FCN	XM_017488985.3	34.63	-12.05	0.00	transmembrane 7 superfamily member 2 (tm7sf2)
FCN	NM_001201135.1	20.26	-4.48	0.01	trans-2,3-enoyl-CoA reductase b (tecrb)
FCN	XM_017457211.3	85.91	-9.37	0.00	ATP citrate lyase a (aclya)
FCN	XM_017469343.3	33.46	-2.35	0.00	3-hydroxyacyl-CoA dehydratase 2 (hacd2)
FCN	XM_017482664.3	56.52	-35.33	0.00	stearoyl-CoA desaturase (delta-9-desaturase) (scd)
FCN	XM_017495847.3	18.06	-2.99	0.01	acyl-CoA synthetase short chain family member 2 like (acss2l)
FCN	XM_053680102.1	13.23	-6.55	0.02	acyl-CoA synthetase long chain family member 2 (acsl2)
NCF	XM_017457211.3	37.34	-9.14	0.00	ATP citrate lyase a (aclya)
NCF	XM_017456076.3	33.58	-3.57	0.00	24-dehydrocholesterol reductase (dhcr24)
NCF	XM_017478833.3	59.78	-4.62	0.00	7-dehydrocholesterol reductase (dhcr7)
NCF	XM_017488985.3	15.31	-4.11	0.03	transmembrane 7 superfamily member 2 (tm7sf2)
NCF	XM_017457211.3	37.34	-9.14	0.00	ATP citrate lyase a (aclya)
NCN	XM_017465598.2	16.09	-3.26	0.01	isocitrate dehydrogenase (NADP(+)) 2 (idh2)
NCN	NM_001201135.1	13.24	-3.53	0.02	trans-2,3-enoyl-CoA reductase b (tecrb)
NCN	XM_017456076.3	12.30	-4.24	0.02	24-dehydrocholesterol reductase (dhcr24)
NCN	XM_017478833.3	19.48	-5.09	0.01	7-dehydrocholesterol reductase (dhcr7)
NCN	XM_017488985.3	18.40	-8.95	0.01	transmembrane 7 superfamily member 2 (tm7sf2)
NCN	NM_001201135.1	13.24	-3.53	0.02	trans-2,3-enoyl-CoA reductase b (tecrb)
NCN	XM_017469343.3	21.75	-2.12	0.01	3-hydroxyacyl-CoA dehydratase 2 (hacd2)
NCN	XM_053680102.1	19.87	-4.57	0.01	acyl-CoA synthetase long chain family member 2 (acsl2)

For energy metabolism, a clear distinction emerged in central carbon metabolism. While not enriched in fed groups, the tricarboxylic acid (TCA) cycle (GO:0006099) was a defining feature of the fasted transcriptome. The mitochondrial enzyme isocitrate dehydrogenase (NADP⁺) 2 (*idh2*) was commonly downregulated in both FCN and NCN. The FCN group showed a broader suppression of TCA-related genes, including 2′,3′-cyclic nucleotide 3′ phosphodiesterase (*cnp*), ATP citrate lyase a (*aclya*), isocitrate dehydrogenase [NAD] subunit γ (LOC108275983), succinate-CoA ligase GDP/ADP-forming subunit α (*suclg1*) and aconitase 2 (*aco2*) (Table 2).

### KEGG pathway analysis supports a fasting-associated immunometabolic phenotype

The Kyoto Encyclopedia of Genes and Genomes (KEGG) enrichment analysis of the consistent DEGs further consolidated the pathway-level differences (Table 3, Table S5). No pathways were significantly enriched across all timepoints in the fed groups. In stark contrast, fasted groups (FCN and NCN) showed persistent enrichment of seven specific pathways at 8 hpc, including valine/leucine/isoleucine degradation (ko00280), fatty-acid elongation (ko00062), steroid biosynthesis (ko00100), tryptophan metabolism (ko00380), DNA replication (ko03030), longevity regulating pathway – worm (ko04212), and Peroxisome Proliferator-Activated Receptor (PPAR) signaling pathway (ko03320). This enrichment profile, particularly the activation of PPAR signaling, a master regulator of lipid metabolism and inflammation, strongly supports a state of catabolic adaptation and immunometabolic stress in fasted fish.

**Table 3:** Significant KEGG pathways (FDR < 0.05) identified only at 8 hpc in the channel catfish liver during vAh challenge.

Trt (time)	Pathway	ID	#Seqs	#Diff. Expr Seqs	GSEA ES	GSEA NES	Fisher FDR
NCN8h	Fatty acid elongation	ko00062	87	13	-0.394	-2.630	0.000
NCN8h	Steroid biosynthesis	ko00100	42	6			
NCN8h	Valine, leucine and isoleucine degradation	ko00280	76	12	-0.450	-2.103	0.038
NCN8h	Tryptophan metabolism	ko00380	111	13	-0.477	-3.019	0.000
NCN8h	DNA replication	ko03030	31	7	-0.286	-1.894	0.003
NCN8h	PPAR signaling pathway	ko03320	87	15	-	-	0.003
NCN8h	Longevity regulating pathway - worm	ko04212	80	12	-	-	0.000
NCN8h					-	-	0.001
FCN8h	Fatty acid elongation	ko00062	87	10			
FCN8h	Steroid biosynthesis	ko00100	42	7	-0.389	-2.445	0.009
FCN8h	Valine, leucine and isoleucine degradation	ko00280	76	14	-	-	0.007
FCN8h	Tryptophan metabolism	ko00380	111	12	-0.508	-3.252	0.000
FCN8h	DNA replication	ko03030	31	5	-0.282	-1.901	0.005
FCN8h	PPAR signaling pathway	ko03320	87	11	-	-	0.036
FCN8h	Longevity regulating pathway - worm	ko04212	80	11	-	-	0.002

### RT-qPCR validation

The expression patterns of a subset of genes including *il-1βa*,* tlr5*,* tecrb*,* dhcr7*,* acsl2*,* hacd2* were independently validated by RT-qPCR using the same RNA samples. The qPCR results showed a high correlation with the RNA-Seq fold-change data (Table S6), confirming the reliability of the sequencing analysis. Although the RT-qPCR validation used a single reference gene, the assay was intended to confirm general concordance with RNA-Seq expression trends rather than serve as a stand-alone quantitative expression analysis.

## Discussion

The liver functions as a pivotal organ, integrating metabolism and immunity, dynamically responding to feed and infection through coordinated tissue and transcriptional responses. This study employed a multi-factorial design and transcriptomics to investigate pre-mortality hepatic response to MAS infection in channel catfish. Our central finding is that feeding status was more associated with early hepatic immunometabolic responses than physical injury under the present experimental and control conditions, while physical injury was associated with additional inflammatory variation. Fasted fish mounted a broad, catabolic response characterized by suppressed lipid anabolism, altered TCA cycle dynamics, and heightened inflammatory signaling. In contrast, fed fish maintained hepatic architecture and exhibited a more restrained transcriptional profile, with a focus on regulated immune signaling and a distinct pattern of sterol metabolic pathway engagement. These results provide a system-level view of how husbandry factors interact to set the hepatic metabolic tone, which in turn may dictate the trajectory of infection outcomes in aquaculture species.

The emphasis on the early post-challenge window (2, 4, and 8 hpc) was intentional, as this interval captures the initial host hepatic response before the later stage at which bacterial burden and overt tissue injury become more pronounced. Although maximal hepatic colonization and more severe pathological manifestations of vAh infection generally occur at later timepoints, the present data show that substantial immunometabolic reprogramming is already underway within the first few hours after challenge. In this context, the early induction of cytokine- and chemokine-associated signaling, together with suppression of lipid biosynthetic pathways and restriction of TCA cycle-associated transcripts, likely represents an early molecular phase of host destabilization that precedes overt structural damage. Thus, the early transcriptional signatures observed here may be viewed as molecular antecedents of subsequent tissue-level deterioration, linking early immunometabolic imbalance to the later vascular and degenerative changes associated with acute MAS.

### Hepatic pathology reflects the metabolic, not inflammatory, cost of infection

The observed histopathology, primarily vascular congestion and edema in the NCN group, aligns with the metabolic dysregulation seen in the transcriptome rather than with direct bacterial damage. The absence of severe necrosis or widespread inflammation within the 8-hour window is consistent with the known pathogenesis timeline of vAh, where hepatic bacterial burden (measured as genome equivalents) typically peaks later (24–48 hpc) [31], suggesting that the direct bacterial presence or the accumulation of cytotoxic factors in the liver was insufficient to induce overt cytopathic changes at this early stage. Interestingly, the most severe histological changes occurred in fasted fish that were not injured (NCN), suggests the early lesions in NCN are likely a consequence of systemic hemodynamic and metabolic response rather than direct bacterial cytopathy. The liver is an immunogenically tolerant organ anatomically equipped to limit the spread of bacterial products through the portal vein; hence, the delayed onset of significant liver lesions pre-mortality suggests that the organ, while central to systemic host defense and metabolic regulation, may initially evade direct cytopathic effects or may possess transient protective mechanisms during early infection [32]. It is worth noting that, despite the absence of overt hepatic lesions, the bacterium can still be isolated from the liver for diagnostic and postmortem confirmation [31, 33]. This finding underscores the limitation of histopathology alone, highlights the value of bacterial quantification and transcriptomics in revealing sub-tissue-level reprogramming that precedes structural change and underscores that mortality in an acute septicemia infection like MAS is a multi-organ process, with each tissue playing different roles [18].

### Feeding status is associated with differences in the extent of the hepatic immune response

The transcriptomic data revealed that the fasted state is associated with an inflammatory response. This primed state is evidenced by a significantly amplified and diversified cytokine and chemokine repertoire in fasted fish, including robust induction of potent pro-inflammatory mediators such as *il1β* and *cxcl9*, beyond the core pathogen-sensing (*tlr5*) and signaling (*il6st*) genes upregulated in all groups. It therefore appears that the fasted liver is poised for aggressive leukocyte recruitment and activation [34–38]; and the fed liver, in contrast, appeared to maintain a more equipped state, engaging specific regulatory pathways (e.g., complement activation in NCF) without unleashing a broad inflammatory cascade. This moderated response in fed fish likely reflects a greater capacity to meet the metabolic demands of immunity without resorting to emergency catabolic programs, thereby preserving tissue homeostasis as seen histologically.

### Metabolic reprogramming: anabolic restraint versus catabolic stress

The most obvious transcriptional differences were observed in metabolic pathways and were most associated with feeding status under the present experimental design. The coordinated downregulation of the acyl-CoA biosynthetic process, particularly in fasted fish, points to a strategic suppression of lipid anabolism and elongation. This may represent a conservation of ATP and reducing equivalents (NADPH) or a pathogen-induced subversion of host lipid metabolism [39, 40]. The concurrent enrichment of PPAR signaling, a regulator of fatty acid catabolism and inflammation, further supports a shift toward lipid mobilization and oxidation in the fasted state [41].

On the other hand, the stark contrast in sterol metabolism is particularly telling. The significant negative enrichment of the cholesterol biosynthesis pathway (*dhcr24*, *dhcr7*, *tm7sf2*) exclusively in fed fish, even amidst global downregulation, indicates a retained, though dampened, anabolic potential. Cholesterol is essential for membrane integrity and cell signaling; its maintained biosynthetic capacity in fed fish could support cellular repair and immune cell function. Its suppression in fasted fish may indicate a severe metabolic compromise or a host strategy to limit lipid resources to the pathogen [42].

Furthermore, the exclusive negative enrichment of the TCA cycle in fasted fish, marked by downregulation of *idh2* and *aco2*, suggests mitochondrial metabolic stress or a purposeful cycling of oxidative metabolism to reduce reactive oxygen species (ROS) production during nutrient scarcity and infection [43, 44]. This constriction of central carbon flux stands in direct contrast to the metabolic phenotype of the fed liver.

### The role of injury on the initiation of inflammation

The present experimental conditions revealed that physical injury was associated with an additional inflammatory component. Fin-clipping was associated with higher numbers of immune-related DEGs and less survival in clipped groups (FCN > NCN; FCF > NCF). This aligns with the established role of epithelial breach in facilitating pathogen entry and activating localized damage-associated molecular pattern (DAMP) signals [17, 45]. Our data suggests that while injury worsens outcomes, it is the underlying metabolic state of the liver, determined by recent feed intake, that establishes the foundational capacity to manage the ensuing infection.

### Experiment limitation

A limitation of this study is that the sham controls were designed to represent fed baseline husbandry conditions with or without adipose fin‑clipping and did not include the reciprocal fasted state. Therefore, the transcriptomic results are best interpreted as infection responses relative to fed baseline conditions (2 hpc), while feeding- and injury-associated differences among infected groups should be viewed as associations observed under these experimental conditions.

## Conclusion

In conclusion, this study shows that early hepatic responses to MAS change under the baseline physiological conditions. Feed deprivation was associated with a more catabolic and inflammatory response profile, whereas recent feeding was associated with a more restrained and metabolically supported response. Physical injury was associated with additional inflammatory changes, but these effects should be interpreted cautiously given the sham control structure. These findings provide a molecular rationale for considering nutritional status in strategies aimed at improving disease resilience in aquaculture.

## Supplementary Information

Supplementary Material 1.

Supplementary Material 2: Supplementary Table 1: RNA integrity and purity metrics of liver samples used for RNA-Seq analysis of channel catfish challenged with virulent *Aeromonas hydrophila*. The table presents the RNA Integrity Number (RIN) and A260:A280 ratio for each liver RNA sample collected at 2, 4, and 8 hours post challenge prior to cDNA library preparation and sequencing.

Supplementary Material 3: Supplementary Table 2: Categorization of differentially expressed genes (DEGs; FDR < 0.05) in the liver of channel catfish (*Ictalurus punctatus*) challenged with virulent *Aeromonas hydrophila*, based on functional association with immune signaling or metabolic processes (carbohydrate, lipid, and protein metabolism). DEGs shown here were consistently differentially expressed across all timepoints (2, 4, and 8 hours post-challenge) within each treatment group.

Supplementary Material 4: Supplementary Table 3: The list of differentially expressed genes (DEGs, FDR < 0.05) in at least one pairwise comparison of channel catfish challenged with virulent *Aeromonas hydrophila*. The liver was sampled at 2, 4 and 8 hours post challenge. FCF: These fish had their adipose fin-clipped and were fed 2 hours before the challenge. FCN: These fish had their adipose fin-clipped and were fasted 2 hours before the challenge. NCF: These fish had intact adipose fins and were fed 2 hours before the challenge. NCN: These fish had intact adipose fins and were fasted 2 hours before the challenge. Control 1: These fish had their adipose fin clipped and were exposed to sterile TSB. Control 2: These fish had intact adipose fins and were exposed to sterile TSB.

Supplementary Material 5: Supplementary Table 4: Gene ontology enrichment using a Fisher’s exact test (adj. *P*-value < 0.05) on the shared DEGs within the treatment groups (FCF, NCF, FCN and NCN); 2-, 4- and 8-hours post challenge. The RNA sequencing libraries were generated from the liver of individual channel catfish challenged with virulent *Aeromonas hydrophila*.

Supplementary Material 6: Supplementary Table 5: The list of enriched Kyoto Encyclopedia of Genes and Genomes (KEGG) derived from differentially expressed genes (DEGs, FDR < 0.05) in the liver of channel catfish challenged with virulent *Aeromonas hydrophila*. The analysis was conducted on DEGs consistently expressed across at least one timepoint (2, 4, or 8 hours post-challenge). Pathway enrichment was evaluated separately for each treatment group using KEGG. Pathways related to lipid metabolism and immune signaling were highlighted.

Supplementary Material 7: Supplementary Table 6: Relative fold-change (FC) values of gene expression in the liver of channel catfish challenged with virulent *Aeromonas hydrophila*. Relative expression was determined through RNA-Seq and reverse-transcription quantitative PCR (RT-qPCR). Six samples from each treatment group at 4 hours post challenge were taken and ran in triplicate to get the mean of each value in the RT-qPCR. For gene validation where the values were either not expressed or had a *p*-value > 0.05, no data (ND) was reported.

## Data Availability

The RNA sequencing datasets generated for this study have been deposited to the NCBI Gene Expression Omnibus (GEO) repository and can be found under accession number GSE280610. All other data that support the findings of this study have been included in the manuscript and Supplementary Materials.
