# Supplementary material for: Hepatic transcriptomics reveals immuno-metabolic interactions in juvenile channel catfish (Ictalurus punctatus) after Aeromonas hydrophila infection
Source: BMC Genomics. 2026 May 28;27:639. doi: 10.1186/s12864-026-12988-1 (PMC13403400; doi:10.1186/s12864-026-12988-1)
Supplement: Supplementary file 1 — Supplementary Material 1. [file 12864_2026_12988_MOESM1_ESM.docx]

Supplementary Material

**Integrated transcriptomics reveals the hepatic immunometabolic interplay between nutrition, physical injury, and *Aeromonas hydrophila* infection in juvenile channel catfish (*Ictalurus* punctatus)**

**Yesutor K. Soku^1,2^, Miles D. Lange^3^, Jason W. Abernathy^3^, Nithin M. Sankappa^3,4^, Craig A. Shoemaker^3^, Karl Hayden^1^, Linnea K. Andersen^3^, Ida Phillips^1,5^, Toufic Nashar^1^, Temesgen Samuel^1^, Abdelrahman Mohamed^1^**

^1^ Department of Pathobiology, College of Veterinary Medicine, Tuskegee University, Tuskegee, Alabama, United States of America

^2^ Department of Biology, College of Arts and Sciences, Tuskegee University, Tuskegee, Alabama, United States of America

^3^ United States Department of Agriculture, Agricultural Research Service, Aquatic Animal Health Research Unit, Auburn, Alabama, United States of America

# Supplementary Figures

D)

C)

B)

A)


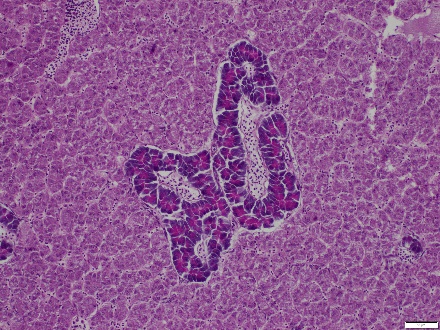

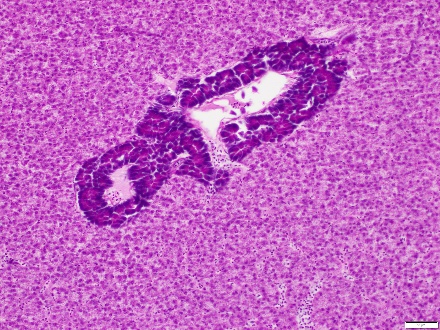

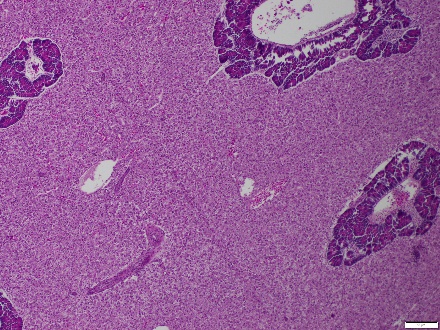

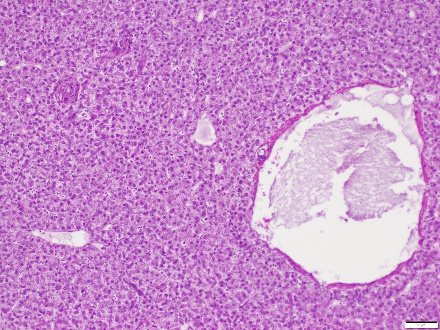


H)

G)

F)

E)


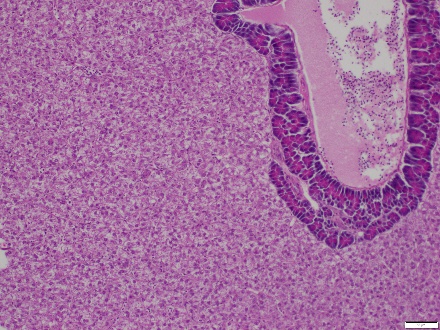

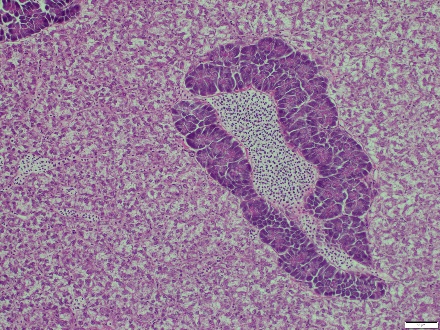

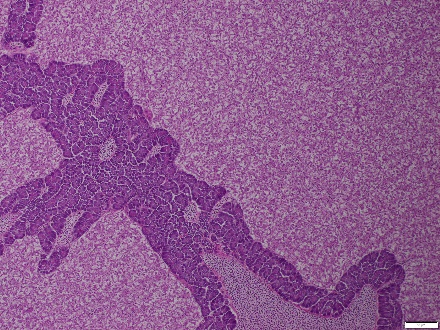

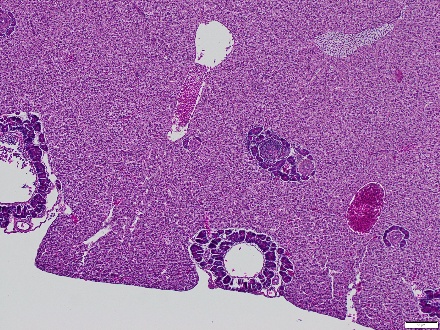


L)

K)

J)

I)


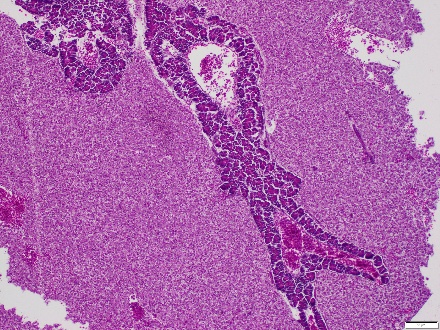

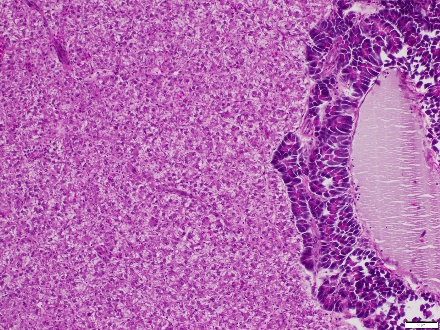

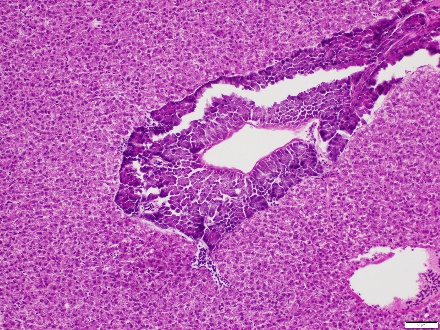

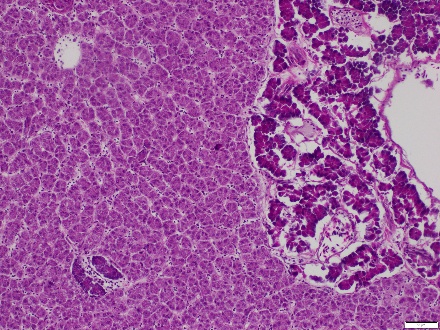


**Supplementary Figure 1:** Photomicrographs of the hepatopancreas of infected channel catfish (*Ictalurus punctatus*) at 2 HPC. (A) Minimal findings, 100x, H&E, FCF. (B) Minimal findings, 100x, H&E, NCF. (C) Minimal findings, 100x, H&E, FCN. (D) Moderate vascular congestion and edema (arrow), 200x, H&E, NCN. At 4 HPC. (E) Minimal findings, 100x, H&E, FCF. (F) Minimal findings, 100x, H&E, NCF. (G) Minimal findings, 100x, H&E, FCN. (H) Moderate vascular congestion, edema, and lymphoplasmacytic infiltration (arrow), 200x, H&E, NCN. At 8 HPC. (I) Minimal findings, 100x, H&E, FCF. (J) Minimal findings, 100x, H&E, NCF. (K) Minimal findings, 100x, H&E, FCN. (L) Moderate vascular congestion, and moderate to severe edema, 200x, H&E, NCN.
